# A Multicomponent Family Treatment of Childhood Obesity Based on the Planetary Healthy Diet: A Randomized Controlled Trial

**DOI:** 10.3390/ijerph22111717

**Published:** 2025-11-13

**Authors:** Joana Maia Brandão, Diana Barbosa Cunha, Magno Conceição Garcia, Cinthia Guimarães Assemany, Marina Campos Araújo, Valéria Troncoso Baltar, Rosely Sichieri

**Affiliations:** 1 Department of Epidemiology, Social Medicine Institute, State University of Rio de Janeiro, Rua São Francisco Xavier, 524, 7° andar, bloco E, sala 7017B, Maracanã, Rio de Janeiro 20550-900, RJ, Brazil; joanamaia24@gmail.com (J.M.B.); magno.ufrrj@gmail.com (M.C.G.); nutrircinthia@gmail.com (C.G.A.); rosely.sichieri@gmail.com (R.S.); 2 Department of Epidemiology and Quantitative Methods in Health, Sergio Arouca National School of Public Health, Oswaldo Cruz Foundation, Rio de Janeiro 20911-300, RJ, Brazil; marina.araujo@fiocruz.br; 3 Department of Epidemiology and Biostatistics, Institute of Collective Health, Fluminense Federal University, Niterói 24030-210, RJ, Brazil; vtbaltar@id.uff.br

**Keywords:** obesity, food consumption, environmental health

## Abstract

The Planetary Health Diet (PHD), recognized as a healthy and environmentally sustainable dietary pattern, has been promoted globally; however, its role in supporting weight change among children within structured weight management interventions remains unclear. A four-month randomized multicomponent family-based trial was conducted with 120 dyads of children with obesity (7–12 years) and their guardians. The intervention group (IG) received counseling on the PHD, portion size reduction, and strategies to increase moderate-to-vigorous physical activity, while the control group (CG) received general guidance based on the Dietary and Physical Activity Guidelines for the Brazilian Population, emphasizing the avoidance of ultra-processed foods. Dietary intake was assessed using a food frequency questionnaire, and anthropometric measurements were taken by trained professionals at baseline and during each consultation. Mixed-effects models were used to estimate BMI change in children and guardians. Most guardians were mothers with low-to-middle income. Attrition was around 40% in both groups, but most participants were followed up for four visits. No significant difference in BMI variation was observed between allocation groups; however, both groups of children reduced BMI (IG = −0.2 and CG = −0.4; *p* = 0.002), with no change among guardians. PHD adherence scores changed minimally overall, but consumption of nuts and fruits increased in both groups. Although the intervention did not outperform the control in reducing BMI, the overall BMI reduction among children in both groups suggests that participation in a lifestyle-focused trial, regardless of specific content, may promote weight management in children with obesity.

## 1. Introduction

Childhood obesity has emerged as an escalating global public health concern. In 2022, more than 390 million children and adolescents aged 5 to 19 were classified as overweight, of whom 160 million were living with obesity [1]. Children in low-and middle-income countries face a dual burden of malnutrition, with undernutrition and overnutrition often coexisting, whereas high-income countries primarily struggle with overnutrition [2]. This situation occurs alongside environmental degradation, a convergence described as the obesity–environmental paradox, highlighting the need for strategies that address both human and planetary health [3]. The Planetary Health Diet (PHD), developed by the EAT-Lancet Commission in 2019, aims to promote healthy diets and sustainable food production by encouraging the consumption of fresh foods and whole grains, minimizing the intake of ultra-processed products, limiting animal-based foods, and using sustainably produced plant-based fats. Such dietary shifts could prevent approximately 11 million deaths per year, representing between 19% and 24% of total adult mortality worldwide [4].

The PHD has since been the focus of scientific debate [5] and adapted for use within the context of local food cultures [6,7]. In Brazil, Marchioni and colleagues developed the Planetary Health Diet Index (PHDI), a scoring system comprising 16 components derived from the EAT-Lancet recommendations. The PHDI accounts for the proportional intake from all food groups and for energy density. Higher PHDI scores have been associated with better diet quality and reduced greenhouse gas emissions [8]. Moreover, it has been inversely associated with obesity in cohort studies. In the ELSA-Brasil cohort, individuals with higher PHDI scores had lower BMI, waist circumference, and a 24% lower likelihood of being overweight or obese [9]. These results suggest that adherence to the PHD may help reduce obesity-related outcomes among adults. In children, adherence to the PHD is associated with a better diet [10], and reduced obesity among children with abdominal obesity [11].

Importantly, clinical trials targeting childhood obesity that focus primarily on weight loss have generally yielded only modest results. These interventions also tend to experience high dropout rates, often because of scheduling conflicts or because they do not fully address the needs and expectations of participating families. In contrast, more comprehensive approaches that promote lifestyle changes involving the entire family have shown greater effectiveness in achieving sustainable health improvements [12,13,14]. In a randomized study conducted with children and adolescents with obesity in Rio de Janeiro, Brazil, combining a dietary plan to reduce ultra-processed food consumption resulted in a greater reduction in BMI compared with guidance focused solely on reducing ultra-processed foods [15].

Portion size moderation, especially for energy-dense foods, is a recommended strategy for weight control [16]. Studies indicate that larger portion sizes are associated with higher BMI in children and adolescents [17], and that the historical increase in portion sizes has led to consumption levels exceeding dietary recommendations [18]. Moreover, parents tend to overestimate the portions served to their children based on their own eating patterns, leading to food intake above children’s energy needs [19]. The shape and size of tableware can also influence food perception and consumption through visual mechanisms, suggesting that utensil design can modulate individual eating behavior [20,21,22].

Thus, the present study aimed to evaluate whether a family-based dietary intervention, grounded in the principles of the Planetary Health Diet and combined with portion size reduction and promotion of physical activity, could reduce excessive weight gain in children with overweight or obesity and their primary caregivers, compared to the control group, which received general guidance on healthy eating and lifestyle habits.

## 2. Materials and Methods

This study involved a two-group parallel non-blinded randomized multicomponent family-based intervention over a period of four months involving children aged 7 to 12 years with overweight or obesity (Z-score ≥ 1.5) accompanied by a guardian. The World Health Organization (WHO) defines overweight as Z-score > +1.0 and obesity as Z-score > +2.0. However, we chose to use a cut-off of Z-score ≥ 1.5, as we considered this threshold closer to the risk range for obesity and it allows for early detection of children at risk [1]. Detailed information on the trial design, methods, and intervention components has been previously published [23]. This study is reported according to the CONSORT Statement for Randomized Trials of Nonpharmacologic Treatments [24], and the corresponding checklist is available as Supporting Information (Table S1).

The dyads of children and one of their guardians were randomized to the intervention or control group. During a period of four months, the intervention arm received counseling based on the PHD adapted to the Brazilian context, combined with portion size reduction strategies, encouragement of regular physical activity, and reduction in sedentary behavior. The guidelines were directed to all family members.

### 2.1. Recruitment and Selection

Eligibility criteria included being within the specified age range, have overweight or obesity and at least one daily meal at home, and not presenting genetic or endocrine disorders related to obesity, conditions preventing accurate anthropometric measurement, current use of weight-loss medication or continuous corticosteroids. These data were self-reported, and nutritional status was assessed at the first in-person visit. Recruitment strategies included outreach in public schools, universities, local radio, and social media. Interested families completed a screening form and attended an in-person session for further explanation and signing of informed consent.

Interested participants could register by emailing their information based on the eligibility criteria. Once selected, participants were scheduled for an initial face-to-face consultation where they received all relevant information about the project. All this information was obtained through a questionnaire completed by the guardian and the child.

### 2.2. Sample Size and Randomization Procedure

The required sample size was calculated as 48 child–guardian dyads in each group, based on an expected difference of 1.72 units in children’s BMI and a standard deviation of 3.0 [25], with 80% statistical power and a 95% confidence level. To account for the substantial dropout rates often reported in clinical trials for childhood obesity interventions, the target enrollment was set at 120 participants, evenly split between the with 60 allocated to each study arm.

Following recruitment, participants meeting the eligibility criteria were randomly assigned to either the intervention or control group. Randomization was conducted in blocks of eight, using a computer-generated random number sequence by the trial coordinator.

### 2.3. Interventions

Participants in the intervention group attended six in-person sessions, the first two spaced 15 days apart, and the remainder monthly, delivered by a multidisciplinary team of nutritionists and physical education professionals at the State University of Rio de Janeiro. They received an educational kit including a recipe booklet, a monthly goal plan, a sticker calendar, three educational activities and family-oriented interactive materials. Each month, children and their families were invited to select one of the following goals: (1) reducing the intake of ultra-processed foods; (2) increasing nut consumption; (3) experimenting with recipes that promote full use of foods; (4) reducing red meat consumption; (5) increasing the intake of whole grains; (6) increasing fruit consumption; and (7) increasing the intake of vegetables and leafy greens. To support adherence, families received a monthly calendar along with stickers to mark the days on which the selected goal was achieved. This playful strategy is designed to engage children and their guardians, encouraging them to follow the recommended guidelines in a motivating and enjoyable manner.

Dietary guidance followed the Brazilian-adapted Planetary Health Diet (PHD), emphasizing plant-based proteins, fresh foods, legumes, fruits, vegetables, whole grains, low-fat dairy, lean meats, eggs, and vegetable oils, while limiting sugar and salt. The PHD was adapted for the traditional dietary habits of Brazilian children and adolescents and to address the country’s socioeconomic diversity and nutritional vulnerabilities. Although the EAT–Lancet Commission recommends reducing red meat for environmental reasons, in low- and middle-income countries such as Brazil, animal-source foods remain critical sources of high-quality protein, iron, zinc, and vitamin B12, which are often insufficient in plant-based diets. According to UNICEF [26], more than half of young children in these contexts do not consume eggs, fish, or meat daily, contributing to widespread micronutrient deficiencies. Thus, the adaptation aimed to maintain nutritional adequacy while promoting gradual and culturally feasible shifts toward more sustainable dietary patterns [23,26].

Portion control strategies included providing children with specially designed utensils to reduce serving sizes, such as shallow plates with colored rims, tall narrow glasses, and small bowls [27]. All participants received the same utensils with standardized guidance, regardless of age or sex, aiming to improve dietary awareness, enhance diet quality, and reduce overall food intake

Participants in the intervention group also received a dance choreography video and two to three gym class links available on YouTube each week to promote moderate-to-vigorous physical activity. On average, the duration of the video classes was 45 min. At the beginning of the intervention, a sports kit containing ropes and balls was provided. To enhance adherence to the intervention, a set of motivational messages was sent weekly via WhatsApp, encouraging children to engage in the activities. Additionally, children and their guardians received a list of goals aimed at reducing sedentary time and increasing physical activity throughout the study period, from which they selected two goals to pursue each week.

All counseling and educational activities targeted both children and their guardians, promoting improvements in the home food environment and reinforcing that changes in eating habits should take place at the family level.

### 2.4. Control Group

The control group received general dietary guidance consistent with current primary care recommendations, which are based on the Dietary Guidelines for the Brazilian Population [28], and physical activity guidance aligned with the Physical Activity Guidelines for the Brazilian Population. During the consultations, discussions with the child and their guardian focused mainly on the “Golden Rule” of the Dietary Guidelines, which emphasizes avoiding the consumption of ultra-processed foods. The control group only participated in discussions about their diet, without encouragement for goal setting, recipe books, or educational activities, and did not receive portion-control utensils.

### 2.5. Data Collection

The participants were recruited from October 2022 to July 2023, and the interventions were conducted from October 2022 to December 2023. Before data collection began, all professionals were properly trained.

The primary outcome of the study was the change in children’s body mass index (BMI). BMI was assessed at all visits, with the first two and the final assessments conducted 15 days apart, and the subsequent visits scheduled at monthly intervals. Body weight and body fat % was assessed using a portable digital scale (Tanita BC-558, Tanita Corporation, Tokyo, Japan), and height was measured twice with a portable stadiometer (Altura Exata, Minas Gerais, Brazil). All measurements were obtained with participants barefoot, dressed in light clothing, free from hair accessories, and standing upright with arms relaxed alongside the body, ensuring alignment to the Frankfurt horizontal plane. Body fat % was assessed using a digital bioelectrical impedance analyzer. The analyzer estimates body fat percentage based on bioelectrical impedance using built-in algorithms adjusted for sex, age, height, and weight. All assessments were performed by trained personnel, with participants instructed to avoid eating, drinking, or engaging in vigorous physical activity for at least four hours before testing, and to empty their bladder immediately prior to measurement. The nutritional status of children and adolescents was determined using BMI-for-age z-scores [29], calculated according to the updated World Health Organization growth reference curves, following the cut-off points for individuals over 5 years of age. This assessment was performed at baseline to confirm eligibility for study participation.

For adults, BMI was calculated by dividing body weight in kilograms by the square of height in meters (kg/m^2^). Classification followed the World Health Organization criteria for individuals aged 18 years or older: underweight, <18.5; normal weight, 18.6–24.9; overweight, 25.0–29.9; obesity class I, 30.0–34.9; obesity class II, 35.0–39.9; and obesity class III, ≥40.0.

All the anthropometric measurements were performed according to standardized procedures described in the Technical Manual for Anthropometric Data Collection of the Brazilian Food and Nutrition Surveillance System [30].

At baseline, information regarding sex, skin color, age, family per capita income, and waist circumference were collected. The waist-to-height ratio (WHR) was calculated by dividing waist circumference (cm) by height (cm). Skin color was self-reported with the options: white, black, and brown. Age was collected in years. Family income was measured by asking: “Considering all monthly incomes in your family, what is the family income in Brazilian currency?”. Followed by the question: “How many people rely on this income?”. These data was reported in Brazilian reais and converted to U.S. dollars (≈5 BRL/USD). Categories were defined in relation to the 2023 Brazilian minimum wage (R$1320 ≈ 264 USD): ≤0.5, 0.5–1.0, 1.0–2.0, and >2.0 minimum wages. This variable was categorized as ‘Up to 132 US dollars’, ‘Between 132 and 264 US dollars’. ‘Between 264 and 528 dollars’ and ‘More than 528 US dollars’. For the measurement of Waist Circumference (WC), a flexible and inelastic tape measure was used, with an amplitude of 150 cm and a variation of 0.1 mm. WC was measured with tape placed horizontally at the midpoint between the lower edge of the last rib and the iliac crest. The measurements were performed with the tape firm on the skin, however, without compression of the tissues, standing with a relaxed abdomen and with arms extended to the side of the body.

### 2.6. Measures of Adherence to Intervention

Food consumption of children was assessed using an adapted version of an FFQ validated for adolescents referring to the past three months, administered at baseline and at the end of the study. Children with the help of the guardian had to report their consumption frequency (“less than once a month or never,” “1 to 3 times per month,” “once a week,” “2 to 4 times per week,” “5 to 6 times per week,” “once a day,” “2 to 3 times per day,” and “four or more times a day.”)The reported consumption frequency for each item was converted into a daily frequency. The option “once a day” was considered equivalent to a daily frequency of 1. Other response options were converted proportionally to this unit. For example, items consumed two to three times per day were assigned a daily frequency of 2.5 [(2 + 3)/2] [31]. The daily intake of each food group (kcal/day) was calculated by multiplying the daily frequency of consumption by the caloric content of standard portions for each food group.

The adapted version of the Planetary Health Diet Index (PHDI) [9] was evaluated. The original PHDI comprises 16 components: nuts and peanuts; legumes; fruits; vegetables; whole cereals; eggs; fish and seafood; tubers and potatoes; dairy; vegetable oils; dark green vegetables to total vegetables ratio; red and orange vegetables to total vegetables ratio; red meat; poultry; animal fats; and added sugars. In our adapted index, 12 components were included, as it was not possible to calculate the intake of vegetable oils, the red and orange vegetables to total vegetables ratio, animal fats, and added sugars due to its absence in the food frequency questionnaire (FFQ). The proportional contribution of each component group to total energy intake was computed (calories from the food group/total calories × 100). Component scores were then assigned, and a composite index was generated, with a maximum possible score of 115 points.

The use of the utensils was assessed at the end of the study through the application of a questionnaire. This instrument investigated whether the child had used the provided utensils, which one was used the most, the frequency of use during the week, as well as the parents’ perception of the amount of food the child consumed, particularly if there was a reduction compared to the beginning of the study.

The weekly physical activity of children and their guardians was measured using triaxial accelerometers (ActiGraph GT3X-BT, Pensacola, FL, USA). These devices were worn on the non-dominant wrist for seven consecutive days, both at the baseline and after the intervention period. The devices were initialized with a sampling rate of 100 Hz. During the period of wear, participants were instructed to refrain from engaging in water-based activities (e.g., swimming in rivers or the sea). For this study, it was deemed necessary that each participant wear the device for a minimum of 16 h per day, with a minimum of three valid weekdays and one valid weekend day required for each participant. The raw acceleration data were processed using the GGIR package (version 3.1-2) in R 4.5.1, with an epoch of 5 s [32]. The acceleration was expressed in milligravity units (mg) and calculated using the so-called ‘Euclidian Norm Minus One’ (ENMO) method. Average Acceleration and Intensity Gradient reflect the daily volume and intensity distribution of PA, respectively [33]. The classification of moderate-to-vigorous physical activity (MVPA) was conducted following the age-specific acceleration thresholds proposed by Hildebrand et al. (2014) [34]. Adherence to the exercise protocol was assessed after the intervention using a self-report questionnaire, in which participants indicated the number of exercise sessions attended per week.

### 2.7. Statistical Analysis

Means and standard deviations for quantitative variables and frequencies (percentages) for qualitative variables were calculated at baseline. Intervention effects were estimated based on intention-to-treat analysis obtained from mixed-effects models using the SAS PROC MIXED (On Demand) procedure. The BMI, PHDI and food groups change models incorporate the terms time, treatment group, and the interaction of time and treatment group. All participants were included in the analysis. Statistical significance was defined as *p* < 0.05.

### 2.8. Ethics and Dissemination

This study was reviewed and approved by the Research Ethics Committee of the Instituto de Medicina Social Hésio Cordeiro, State University of Rio de Janeiro (CAAE 59008422.8.0000.5260, approved on 1 September 2022. This is an IRB (Institutional Review Board). Informed consent was obtained from all participants and their legal guardians by signing the consent form of guardians and children’s consent, pursuant to Resolution nº 466/2012.

The study protocol was registered in the virtual platform of free access for registration of experimental and non-experimental studies conducted in humans and conducted in Brazilian territory, the Brazilian Registry of Clinical Trials (RBR-10 mm62vs). Registered 10 February 2023.

## 3. Results

**Figure 1:**
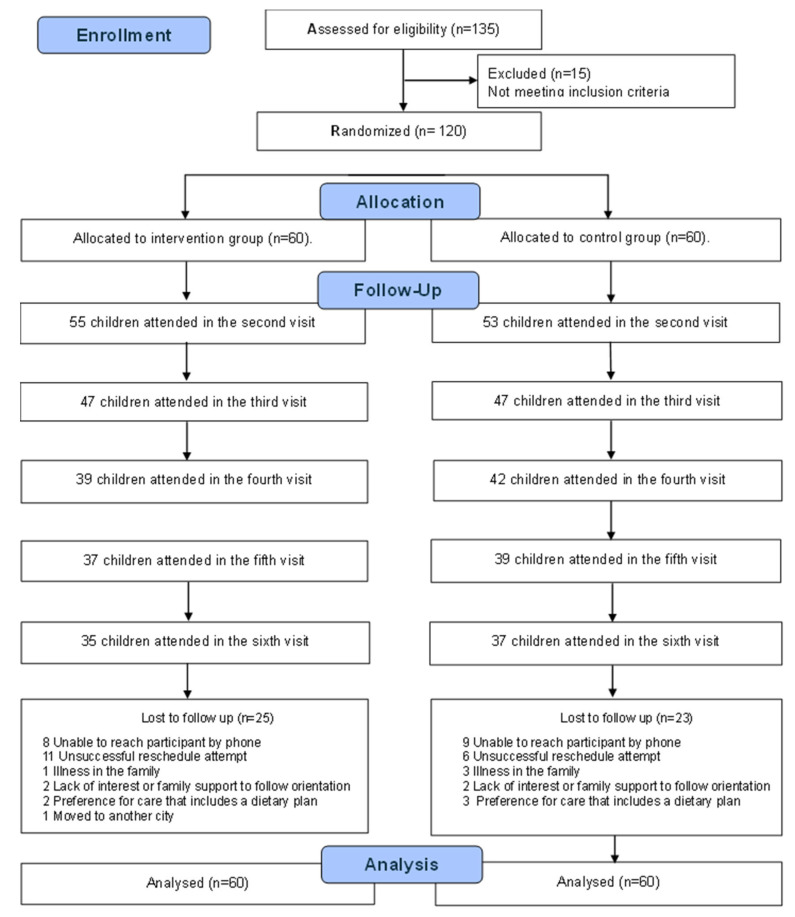
Flow diagram of the progress of subjects through the study phases.

The final sample of this trial consisted of 120 dyads of children and their guardians, with 60 allocated to the intervention group and 60 to the control group. Fifteen children assessed for eligibility did not meet the inclusion criteria. At the end of the study, loss to follow-up was 41.7% in the intervention group and 38.3% in the control group. However, 65% and 72% of the children contributed at least four measurements in the intervention and control groups, respectively. Figure 1 presents the allocation and follow-up losses for each group at each assessment point, indicating that attrition occurred in a similar and balanced manner across both randomized groups.

At baseline, groups’ characteristics were quite similar, with 60% female, a mean age of 9 years, about 32% identified as white, and most classified as physically inactive. Regarding weight status, most children in the intervention group were classified with obesity, while those in the control group were predominantly classified with severe obesity. However, the mean BMI was similar between the groups (Table 1).

In relation to physical activity, time in moderate-vigorous activity was similar between the groups, with the control group engaging in slightly more activity. Despite this, the mean acceleration and intensity gradient were similar, demonstrating a similar accumulation pattern between the groups. Of the 60 participants in the intervention group, 31 responded to the questionnaire about adherence to the physical activity intervention, and 15 showed minimal participation. Therefore, 25% of the sample adhered to the intervention (Table 1).

Most guardians in both groups were female with overweight or obesity, with a mean age of 41 years. The mean BMI was similar between the groups (31.3 kg/m^2^ in the intervention group and 32.0 kg/m^2^ in the control group). More than 50% had a mean monthly income below 264 US$ (Table 1).

Children in the intervention group showed a BMI reduction of 0.2 kg/m^2^, while those in the control group had a reduction of 0.4 kg/m^2^. There was no statistically significant difference between the groups (time * treatment interaction effect, *p*-value = 0.08), but both groups had a reduction in BMI (*p*-value of time = 0.02) (Table 2). Concerning waist circumference, the control group demonstrated a mean reduction of 0.68 cm, whereas the intervention group exhibited a greater decrease of 2.49 cm; however, the between-group difference did not reach statistical significance (*p* = 0.19). With respect to body fat percentage, the control group showed a mean reduction of 0.64%, while the intervention group experienced a slight increase of 0.57%, with no significant time × treatment interaction (*p* = 0.34).

As for the guardians, the intervention group experienced an increase of 0.1 kg/m^2^, while the control group had an increase of 0.2 kg/m^2^, with no statistically significant difference between the groups (*p*-value for time × treatment effect = 0.84).

**Figure 2:**
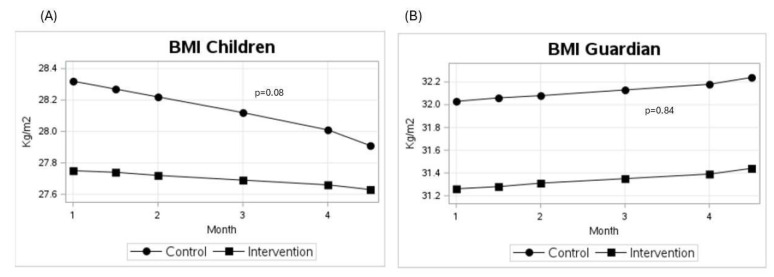
Estimated mean in BMI over time for children (**A**) and Guardian (**B**) by allocation group. *p*-value = time × treatment.

The estimated mean BMI over time for children (A) and Guardian (B) by allocation group is shown in Figure 2.

There was no difference between groups in the index of PHD change over time (*p* = 0.80), as shown Table 3. However, few items had changed in both groups, such as fruits, nuts and peanuts, red meat, chicken and ultraprocessed food (Table 3).

At the end of this study the use of utensils to reduce the portions was inquired and 66.6% of participants in the intervention group reported utilizing the utensils daily (7 times per week), 16.6% indicated usage on nearly a daily basis (5–6 times per week), 3.3% reported moderate usage (3–4 times per week), and 3.3% reported minimal usage (1–2 times per week). No participants indicated never using the utensils, and 10% of participants did not provide a response to the questionnaire. No adverse events were observed by our staff or reported by the family.

Table 4 presents the comparison of baseline characteristics between participants who completed all phases of the RCT and those who discontinued participation. A higher proportion of girls dropped out of the trial (71% vs. 48%, *p* = 0.01), indicating a statistically significant difference in sex distribution between completers and dropouts and participants who completed all phases of the trial were slightly older than those who dropped out (mean age 9.7 vs. 9.0 years, *p* = 0.02), indicating a statistically significant difference in age between groups.

## 4. Discussion

In this multicomponent family intervention, adapted from the PHD to the Brazilian context, both groups experienced reductions in BMI, with no additional decrease in the intervention group compared with the control group. Monthly appointments in both groups were associated with small decreases in BMI and energy intake. Considering that participants were still in a growth phase, reductions in BMI over time in both groups indicate that participation in a structured program focused on healthy lifestyle behaviors may influence this outcome. This effect occurred despite the more intensive guidance provided to the intervention group, which included dietary recommendations, physical activity strategies, and the use of portion-control utensils.

The PHDI in both groups of about 50 in a total score of 115 indicates a low adherence to the PHD at the beginning and end of the study; however, items with small statistically significant changes included an increase in fruits, nuts, and chicken, and a reduction in red meat and ultra processed foods. Particularly, the difference in red meat was significant, with the control group showing great intake. Most guidelines for the prevention and treatment of childhood obesity include the reduction in ultra-processed foods, red meat, and sugar-sweetened beverages. The PHD, in turn, emphasizes the consumption of plant-based foods, whole grains, and nuts. In our study, only nut intake showed a slight increase, while vegetable and whole grain consumption remained unchanged.

The low adherence to the Planetary Health Diet observed in both groups can be explained by multiple interrelated factors. In Brazil, the habitual diet is characterized by high consumption of ultra-processed foods and low intake of whole grains, fruits, and vegetables, while the relatively high cost of fresh and minimally processed foods represents a structural barrier—especially among low-income families. Hirvonen et al. [35] showed that Latin America and the Caribbean have the highest cost for the PHD, exceeding the average daily per capita income of millions of people in low- and middle-income countries. Similarly, Miller et al. (2016) [36] indicated that low fruit and vegetable intake is linked to difficulties in affording these foods, and Caldeira et al. (2025) [37], analyzing Brazilian data, found low adherence to the PHD and an inverse association between the EAT-Lancet score and diet cost. In our sample, about half of the participants reported a per capita family income of approximately US$264, only slightly above the national minimum wage, suggesting substantial financial barriers to following the PHD. Beyond economic limitations, cultural and behavioral factors, along with limited parental engagement, may have further hindered dietary changes over time. These findings highlight the need to tailor future interventions to the socioeconomic context, increase professional support, and actively involve parents to promote long-term adherence.

In Brazil, the prevalence of ultra-processed food consumption among children aged 0–5 years is very high (93.0% in the previous ENANI-2019). Furthermore, 27.4% of children do not consume fruits and vegetables [38]. Among adolescents, data from the Household Budget Survey (POF) indicate that ultra-processed foods account for 26.5% of total caloric intake [39]. These figures help explain the low adherence to the Planetary Health Diet at baseline and also during the study. As a positive finding, although a previous study conducted in Rio de Janeiro among children [15] indicated that an individualized dietary plan with reduced intake of ultra-processed foods may yield greater weight loss, our study observed small yet consistent and clinically meaningful reductions in body weight across both groups. This finding suggests that interventions implemented within primary health care settings can be effective, even when based solely on general dietary advice. Notably, the control group, despite receiving only general guidance, exhibited modest improvements in food group consumption and a significant reduction in total energy intake, comparable to the intervention group. Importantly, the general guidance provided in this study was grounded in the Dietary Guidelines for the Brazilian Population, and a qualitative counseling approach based on these principles demonstrated a promising effect.

Similarly to our results, a study conducted with data from various countries in Latin America (Latin American Study of Nutrition and Health 2016), including Argentina, Brazil, Chile, Colombia, Costa Rica, Ecuador, Peru and Venezuela, have shown limited alignment with the recommended dietary patterns of the Planetary Health Diet (PHD) proposed by the EAT-Lancet Commission. On average, the PHD adherence by country did not reach 50% of the scale. The participants’ ages were between 15 and 65 years old. The higher the age, the higher the PhD adherence; however, less than half of the scale [40]. In 2016, with data from the Portuguese National Dietary Survey, the IAN-AF 2015–2016, the adults over 18 years, showed adherence of only 36.0 (CI95: 35.4; 36.6) [31].

Numerous studies have investigated the impact of multicomponent family-based interventions on children’s BMI. A randomized controlled trial evaluating a family-based childhood obesity prevention program found no significant association between the intervention and BMI reduction. Nonetheless, even modest changes in BMI may be linked to improvements in certain metabolic biomarkers among obese youth [41]. Naik et al. (2020) [42] reported that BMI stabilization following six months of lifestyle intervention was associated with reductions in visceral adiposity and improved physical fitness in obese children, even in the absence of significant changes in total body fat. Systematic reviews have been published on the subject, reporting modest or no effects on BMI in the short, medium, and long term [43,44].

The discrepancy between the weight reduction observed in children and the weight gain among parents may reflect differences in focus and adherence to the intervention. Although adults also received guidance on diet and physical activity, the program was primarily centered on the children, who were the only ones to receive portion-control utensils, a factor that may have promoted greater engagement within this group. In contrast, adults face multiple barriers to weight management, such as entrenched eating habits, emotional or stress-related eating, lower metabolic rates, lack of family support, increased food intake from leftovers, and time constraints due to domestic and occupational demands [45]. Moreover, socioeconomic constraints may lead parents to prioritize healthier foods for their children while maintaining less adequate dietary patterns for themselves [46].

A randomized controlled trial aimed at evaluating the effect of a family-based lifestyle intervention focusing on diet and physical activities on the BMI of children obtained similar results, with no significant difference in BMI between the groups. The researchers suggest that both stabilization and a modest reduction in BMI (which would tend to naturally increase as children grow) in both groups indicate that simply participating in a study focused on healthy lifestyle behaviors can act as a significant incentive for lifestyle change [47].

Clinical trials with children and adolescents using an intervention based on the PHD are practically nonexistent, with most studies being observational [10,40,48]. The only study found refers to a Spanish randomized controlled clinical trial that evaluated changes in adherence to the PHD score and PHD index after a family-based lifestyle intervention among 107 children and adolescents, aged 7 to 16 years, with abdominal obesity. The authors found that after the initial phase of an 8-week intensive intervention based on a moderately hypocaloric Mediterranean diet combined with nutritional education and encouragement of physical activity, there was a significantly improved adherence to the PHD score and index, notable reductions in BMI, weight, waist and hip circumference, and clinical outcomes, such as reductions in glucose and total cholesterol levels [11].

A systematic review evaluating the relationship between portion size and adiposity indices in children found, among other considerations, that it remains unclear whether portion size directly contributes to increased adiposity, whether children with higher adiposity (possibly due to hereditary predisposition) tend to consume larger quantities of food, or whether these associations are explained by other factors that precede food intake [49]. In our study, the intervention group showed high adherence to the utensils, and it was expected that by reducing portion size, this group would present a greater reduction in energy intake compared with the control group. However, we observed the opposite.

Considering adherence-related challenges, some limitations of the study should be acknowledged. Substantial losses to follow-up, the short duration of the intervention, and adaptations made to the PHDI due to missing information in the FFQ may have reduced the validity of the index. Nonetheless, the analysis of individual items provided useful insights, offering results that are more directly applicable to potential dietary recommendations than the composite index.

The strength of this study lies in the finding that even general counseling for individuals seeking treatment, as opposed to a multicomponent family-based intervention, was effective for weight loss. Attrition bias represents a potential limitation of this study, as participants who discontinued the intervention differed from those who completed it, particularly in terms of sex distribution. Such differential loss to follow-up could have influenced the internal validity of the findings. Nevertheless, we employed mixed-effects models, which appropriately handle unbalanced data and missing observations under the missing-at-random assumption. This analytical approach allows the use of all available data and reduces, though does not completely eliminate, the potential impact of attrition on the study outcomes.

## 5. Conclusions

This multicomponent family-based intervention demonstrated that structured programs promoting healthy lifestyles can support reductions in BMI among children. Although no statistically significant effect was observed, this study represents a step forward by integrating human and environmental health perspectives and provides valuable insights for future interventions. Public policies incorporating school or community components could further strengthen home-based actions.

## Figures and Tables

**Table 1:** Baseline characteristics of the participants.

	Children		Adults	
	Intervention (n = 60) n (%)	Control (n = 60) n (%)	Intervention (n = 60) n (%)	Control (n = 60) n (%)
Sex				
Female	36 (60)	35 (58)	55 (92)	57(95)
Skin Color				
White	19 (32)	20 (33)	20 (34)	20 (34)
Black	12 (20)	17 (28)	17 (28)	17 (28)
Brown	29 (48)	23 (39)	23 (38)	23 (38)
BMI category				
Normal Weight	0	0	8 (14)	6 (10)
Overweight	6 (10)	2 (3)	16 (28)	19 (33)
Obesity	28 (47)	22 (37)	19 (33)	16 (28)
Severe Obesity	26 (43)	36 (60)	14 (25)	17 (29)
Monthly family per capita Income *				
Up to $132 dollars	14 (23)	15 (25)		
Between $132 and 264 dollars	19 (32)	16 (27)		
Between $264 to 528 dollars	16 (27)	17 (28)		
More than $528 dollars	11 (18)	12 (20)		
	Mean (SD)	Mean (SD)	Mean (SD)	Mean (SD)
BMI (Kg/m^2^)	27.8 (4.7)	28.3 (5.2)	31.3 (5.6)	32.0 (6.6)
% Body Fat	37.3 (6.9)	38.7 (7.5)	38.2 (8.9)	39.6 (6.7)
Age (y)	9.7 (1.5)	9.1 (1.6)	41.7 (6.5)	41.7 (7.0)
WC (cm)	82.6 (10.3)	84.4 (11.6)	88.6 (15.1)	92.7 (15.1)
WHR	0.56 (0.06)	0.58 (0.06)	0.57 (0.09)	0.54 (0.09)
PHDI	51.3 (7.1)	50.2 (5.2)		
Physical Activity a, b				
Average Acceleration	35.2 (7.2)	36.9 (9.1)	25.8 (6.3)	26.8 (7.1)
Intensity Gradient	−2.19 (0.1)	−2.14 (0.1)	−2.59 (0.1)	−2.58 (0.2)
Moderate-vigorous Physical activity	45.2 (17.3)	50.5 (20.5)	85.3 (29.4)	90.0 (44.6)

BMI—Body Mass Index; WC—Waist Circumference; WHR—Waist-to-Height Ratio; PHDI—Planetary Health Diet Index; a: children with valid accelerometer data: 22 control; 17 intervention; b: guardians with valid accelerometer data: 22 control; 22 intervention; * Poverty line equal to $3.00 per person per day.

**Table 2:** Body Mass Index (BMI) crude means and estimated change (Δ) from baseline by allocation group.

	Baseline		C2 *		C3		C4		C5		C6		Δ		*p* **	*p* ***
	Mean (SD)															
	IG n = 60	CG n = 60	IG n = 55	CG n = 53	IG n = 46	CG n = 46	IG n = 39	CG n = 40	IG n = 36	CG n = 35	IG n = 31	CG n = 33	IG	CG		
BMI (Kg/m^2^) Children	27.8 (4.7)	28.4 (5.2)	27.8 (4.7)	28.4 (5.4)	27.5 (4.6)	28.2 (5.7)	27.6 (4.6)	28.4 (6.0)	27.4 (4.8)	28.2 (5.9)	27.3 (4.9)	28.2 (5.9)	−0.1	−0.4	0.002	0.08
BMI (Kg/m^2^) Guardian	31.3 (5.6)	32.2 (6.6)	31.4 (5.5)	32.2 (6.5)	30.8 (5.2)	32.4 (6.7)	31.6 (5.3)	32.8 (6.7)	31.5 (5.7)	33.39 (6.9)	31.19 (5.6)	33.49 (7.1)	+0.2	+0.2	0.009	0.84

* C—Clinic Visit; ** *p*-value for time,*** *p*-value time × treatment.

**Table 3:** Crude and estimated means in children’s PHDI and estimated means in caloric percentage from food groups consumed by children, stratified by allocation group.

Food Groups	IG		CG		*p*-Value Time	*p*-Value Time × Treatment
	Baseline	End of Study	Baseline	End of Study		
Crude PHDI	50.27 (7.1)	51.30 (6.0)	50.17 (5.2)	50.20 (5.1)		
Estimated PHDI	51.30	51.00	50.17	50.26	0.91	0.82
Nuts and Peanuts	0.6	1.3	0.7	1.2	<0.01	0.67
Legumes	3.8	4.4	4.0	5.0	0.068	0.54
Fruits	4.3	4.1	3.0	5.1	0.033	0.013
Whole grains	2.6	3.3	3.3	5.4	0.099	0.42
Eggs	2.5	2.3	2.8	2.8	0.89	0.83
Fish and Seafood	0.4	0.5	0.4	0.4	0.61	0.60
Tuber and Potatoes	1.7	2.5	2.2	2.1	0.17	0.12
Dairy	13.6	14.2	12.7	13.0	0.68	0.87
Dark Green Vegetables	0.9	0.9	0.7	0.9	0.37	0.65
Vegetables	2.4	2.4	2.6	3.8	0.22	0.17
Read Meat	2.2	2.2	1.7	3.5	<0.001	<0.001
Chicken	2.1	2.4	2.1	2.7	0.027	0.49
Ultraprocessed Food	34.0	27.3	33.5	22.5	<0.001	0.19
Total Energy	2489	2046	2518	1604	<0.001	0.061

**Table 4:** Baseline characteristics of the participants who completed and dropout the trial.

	Children			Guardian		
	Completed (n = 64) n (%)	Dropout (n = 56) n (%)	*p*-Value	Completed (n = 62) n (%)	Dropout (n = 58) n (%)	*p*-Value
Sex			0.01			0.2
Female	31(48)	40 (71)		56 (97)	56 (90)	
Skin Color			0.2			0.4
White	25 (39)	14 (25)		21 (36)	19 (31)	
Black	16 (25)	13 (23)		19 (33)	15 (24)	
Brown	23 (36)	29 (52)		18 (31)	28 (45)	
BMI category			0.08			0.8
Normal Weight	0	0		6 (11)	8 (14)	
Overweight	5 (5)	3 (5)		18 (32)	17 (29)	
Obesity	32 (50)	18 (32)		16 (29)	19 (33)	
Severe Obesity	27 (42)	35 (63)		17 (30)	14 (24)	
Family Income per capita						
Up to $132 dollars	13 (20)	16 (29)	0.6			
Between $132 and 264 dollars	19 (30)	16 (29)				
Between $264 a 528 dollars	21 (33)	12 (21)				
More than $528 dollars	11 (17)	12 (21)				
	Mean (SD)	Mean (SD)		Mean (SD)	Mean (SD)	
BMI (Kg/m^2^)	28.0 (5.3)	28.1 (4.7)	0.9	32.1 (6.5)	31.3 (5.8)	0.5
% Body Fat	37.3 (7.0)	38.4 (7.4)	0.4	38.7 (7.4)	39.1 (8.4)	0.8
Age (y)	9.7 (1.6)	9.0 (1.5)	0.02	43.0 (6.9)	40.5 (6.3)	0.03
WC(cm)	83.8 (11.4)	83.2 (10.4)	0.8	91.7 (13.7)	89.7 (16.6)	0.5
PHDI	50.4 (6.6)	51.1 (5.7)	0.6	116.4 (18.6)	118.7 (18.0)	0.5
Physical Activity a, b						
Average Acceleration	35.9 (8.7)	36.3 (8.1)	0.8	25.3 (6.4)	26.8 (6.9)	0.4
Intensity Gradient	−2.16 (0.1)	−2.16 (0.16)	0.9	−2.60 (0.18)	−2.58 (0.1)	0.5
Moderate-vigorous Physical activity	48.6 (20.9)	47.8 (17.8)	0.8	83.2 (35.6)	90.2 (38.7)	0.8

a: children with valid accelerometer data: 22 control; 17 intervention; b: guardians with valid accelerometer data: 22 control; 22 intervention.

## Data Availability

Data supporting the findings of this study are available from the corresponding author upon reasonable request.

## Supplementary Materials

The following supporting information can be downloaded at: https://www.mdpi.com/article/10.3390/ijerph22111717/s1, Table S1: Checklist of Items Recommended by the CONSORT 2010 Statement.
